# Searching for ‘the method’ in the assessment of complex mitral valve

**DOI:** 10.1186/s44348-024-00004-7

**Published:** 2024-07-30

**Authors:** Ran Heo

**Affiliations:** Division of Cardiology, Hanyang University Medical Center, Hanyang University College of Medicine, 222 Wangsimni-Ro, Seongdong-Gu, Seoul, 04763 Korea

See the article "Two-Dimensional Transthoracic Measure of Mitral Annulus in Mitral Valve Prolapse and Moderate to Severe Regurgitation: A Method Comparison Analysis With Three-Dimensional Transesophageal Echocardiography" in volume 32 on page e2.

Assessing mitral regurgitation (MR) requires an in-depth investigation of both the structure and function of the mitral valve (MV). In clinical practice, two-dimensional echocardiography (2DE) is a primary tool in the initial diagnosis and follow-up of MR.

The pulse-wave Doppler flow technique, which combines pulsed wave Doppler with measurements of the mitral annular (MA) and left ventricular outflow tract (LVOT) areas, allows for the calculation of regurgitant volume and fraction. However, the assumption of MA as a circle could lead to miscalculation as it is an elliptical structure.

In this issue of the *Journal of Cardiovascular Imaging*, Berthelot-Richer et al. [1] explored various methods to assess the MA in subjects with MV prolapse and moderate to severe MR, utilizing three-dimensional echocardiography (3DE) as a reference. Contrary to the authors’ hypothesis and the anatomy of MA, the circular MA model with a diameter derived from an apical 4-chamber view resulted in the least underestimation of the MA. It could be linked to the variability in MA diameter measurements from the apical 2-chamber view, as postulated by the authors.

Several research groups have also reported underestimated MA diameters through 2DE. Anwar et al. [2] reported a discrepancy in MA diameter measurements between 2 and 3DE or cardiac magnetic resonance imaging (CMR), with 3DE measurements exhibiting closer alignment with those obtained from CMR. Similarly, Hyodo et al. [3] observed that the MA diameters obtained from conventional apical 2- or 4-chamber views inadequately represented the actual dimensions of the MA.

Considering the limitations of 2DE, there has been growing interest in 3DE to disclose the pathophysiology, structure, and quantitation of MR. Three-dimensional transesophageal echocardiography offers a more precise visualization of MV morphology, individual scallops, leaflet variations, and commissures [4, 5]. Commercially available 3DE software options facilitate high-resolution reconstruction of the MV with the tracking of landmarks in the whole cardiac cycle to approach the change of annulus and leaflets [6–10]. Moreover, 3DE has exhibited its utility in MR quantification. The three-dimensional proximal isovelocity surface area (PISA) method demonstrated superior accuracy in MR quantification compared to the conventional two-dimensional PISA approach, particularly in cases of eccentric MR and an orifice with asymmetry [11]. Various MR analyses with 3DE techniques have shown closer agreement with those from CMR than the two-dimensional PISA method [12]. It also enables to overcome the limitation of 2DE assessment in the cases of multiple MR jets [12, 13].

With the evolution of 3DE technology, evaluating the MA through three-dimensional transthoracic echocardiography has become available and precise with less variability. The 3DE also demonstrated concordance between results from transthoracic and transesophageal echocardiography [9]. This advancement is especially relevant with the introduction of transcatheter MV interventions. Precise assessment of MA in transcatheter therapies is essential, as some of them target annular repair. Even in transcatheter edge-to-edge repair, the dynamics of MA could be helpful in its planning and execution.

Computed tomographic analysis of annular dimensions in MR mirrors 3DE analysis [14] and holds significance in the preparation of transcatheter MV interventions, by relating MA to LVOT [14–16] to prevent LVOT obstruction and identify calcifications.

In conclusion, while the refinement of 2DE assessments of MR remains essential, the findings from the current issue underscore the significance of incorporating complementary imaging such as 3DE, computed tomography, or CMR. Looking ahead, 3DE has the potential to uncover factors related to MV characteristics that influence intervention success and the absence of residual MR. To facilitate future applications, expediting the analysis of 3DE dynamic MV characteristics through semiautomatic or automatic methods is crucial for seamless integration into clinical practice.
